# GlyNAC (Glycine and N-Acetylcysteine) Supplementation in Mice Increases Length of Life by Correcting Glutathione Deficiency, Oxidative Stress, Mitochondrial Dysfunction, Abnormalities in Mitophagy and Nutrient Sensing, and Genomic Damage

**DOI:** 10.3390/nu14051114

**Published:** 2022-03-07

**Authors:** Premranjan Kumar, Ob W. Osahon, Rajagopal V. Sekhar

**Affiliations:** Translational Metabolism Unit, Section of Endocrinology, Diabetes and Metabolism, Department of Medicine, Baylor College of Medicine, Houston, TX 77030, USA; premranjan.kumar@bcm.edu (P.K.); ob.osahon@bcm.edu (O.W.O.)

**Keywords:** GlyNAC, lifespan, oxidative stress, oxidant damage, mitochondria, mitophagy

## Abstract

Determinants of length of life are not well understood, and therefore increasing lifespan is a challenge. Cardinal theories of aging suggest that oxidative stress (OxS) and mitochondrial dysfunction contribute to the aging process, but it is unclear if they could also impact lifespan. Glutathione (GSH), the most abundant intracellular antioxidant, protects cells from OxS and is necessary for maintaining mitochondrial health, but GSH levels decline with aging. Based on published human studies where we found that supplementing glycine and N-acetylcysteine (GlyNAC) improved/corrected GSH deficiency, OxS and mitochondrial dysfunction, we hypothesized that GlyNAC supplementation could increase longevity. We tested our hypothesis by evaluating the effect of supplementing GlyNAC vs. placebo in C57BL/6J mice on (a) length of life; and (b) age-associated GSH deficiency, OxS, mitochondrial dysfunction, abnormal mitophagy and nutrient-sensing, and genomic-damage in the heart, liver and kidneys. Results showed that mice receiving GlyNAC supplementation (1) lived 24% longer than control mice; (2) improved/corrected impaired GSH synthesis, GSH deficiency, OxS, mitochondrial dysfunction, abnormal mitophagy and nutrient-sensing, and genomic-damage. These studies provide proof-of-concept that GlyNAC supplementation can increase lifespan and improve multiple age-associated defects. GlyNAC could be a novel and simple nutritional supplement to improve lifespan and healthspan, and warrants additional investigation.

## 1. Introduction

Factors responsible for increasing length of life are not well understood. Although there is a growing quest to slow aging via drug discovery [1], identifying effective interventions to increase lifespan remain a challenge. Clues to unravel the mystery of longevity come from factors identified as contributors to the biology of the aging process such as elevated oxidative stress (OxS) and mitochondrial dysfunction, which were proposed decades ago as cardinal theories of aging [2,3]. Elevated OxS is inherently toxic to cellular and organ health [4,5,6,7,8,9,10,11,12,13,14,15,16,17,18,19], and its damaging potential is further amplified by the deficiency of the endogenous antioxidant glutathione (GSH). More recent identification of ‘hallmarks of aging’ [20] include mitochondrial dysfunction, which results in decreased energy generation and adversely affects cellular processes which require energy for their sustained function [21,22,23,24], abnormalities in mitophagy and nutrient sensing and genomic damage [20]. GSH is the most abundant endogenous intracellular antioxidant tripeptide composed of glycine, cysteine and glutamic acid. GSH performs a critically important role of protecting cells from toxic OxS [25,26,27], and we discovered and reported the GSH adequacy is critically necessary for optimal mitochondrial fuel oxidation [28]. GSH levels tend to decline with increasing age in parallel with an increase in age-associated increase in OxS and mitochondrial dysfunction. We also discovered and reported that supplementing GlyNAC (glycine and N-acetylcysteine, as a cysteine donor) in human trials corrected GSH deficiency, OxS and mitochondrial dysfunction, and in HIV-patients it also improved mitophagy, nutrient sensing and lowered genomic damage, and reversed premature aging [29,30]. We coined the phrase ‘*the power of three*’ to indicate that improvements occurred due to the combined effects of glycine, NAC and GSH. Because GlyNAC supplementation improves multiple key defects associated with human aging, we hypothesized that GlyNAC supplementation could improve longevity by improving GSH deficiency, OxS, mitochondrial dysfunction, abnormalities in mitophagy and nutrient sensing, and genomic damage at the organ level. We tested our hypotheses in wild-type C57BL/6J mice by evaluating the impact of GlyNAC supplementation in two parallel studies (a) on length of life, and (b) on GSH synthesis and concentrations, OxS, mitochondrial function, mitophagy, nutrient sensing and genomic damage in the heart, liver and kidneys. The results of our studies are reported in this manuscript.

## 2. Materials and Methods

### 2.1. Mouse Studies

Rodent study protocols were approved by the Institutional Animal Care and Use Committee, and adhered to the criteria outlined in the ‘Guide of the Care and Use of Laboratory Animals’.

### 2.2. Study 1: The Effect of GlyNAC Supplementation on Longevity

32 C57BL/6J mice (16 male; 16 female) were purchased (The Jackson Laboratory, Sacramento, CA, USA), and housed and aged in a dedicated animal satellite facility (with temperature and light control) with daily attention to health and well-being. All mice were fed a regular diet *ad libitum* (protein 25.6%, 3.0 kcal/g feed; Harlan Teklad, Indianapolis, IN, USA). At the age of 65-weeks, one group of 16 mice (8 male, 8 female) were switched to a diet supplemented with glycine and N-acetylcysteine (protein 23.5%, 3.0 kcal/g feed, N-acetylcysteine (NAC) 1.6 mg/g feed and glycine 1.6 mg/g feed; Harlan Teklad, Indianapolis, IN, USA) *ad libitum.* The GlyNAC-supplemented diet was isocaloric and isonitrogenous to the regular diet consumed by the control group of 16 mice (eight male; eight female). Thus, both groups of mice received identical calories and similar protein nitrogen intake per day. These doses of supplemental glycine and NAC were chosen to approximate doses of GlyNAC used in clinical trials in humans [29,30,31]. The mice were fed their assigned diets for the duration of their natural lives, and length of life was recorded. No interventions or studies were performed on these mice in order to avoid stress.

### 2.3. Study 2: The Effect of GlyNAC Supplementation on Key Age-Associated Defects in the Heart, Liver and Kidneys

This separate and parallel study was conducted to investigate mechanisms which could have contributed to longevity as a result of GlyNAC supplementation. Fifteen young C57BL/6J male mice were purchased (The Jackson Laboratory, USA) and housed and aged in a dedicated rodent satellite facility (with temperature and light control) and daily monitoring of animal health. One group of mice served as young controls (Y, *n* = 5), and received the regular diet (as described above) for eight-weeks from the age of 20-weeks and were euthanized. The remaining 10 mice were aged in the satellite until they reached 90-weeks of age, and were then studied in two groups, as old controls (OC, *n* = 5), and as old mice receiving a GlyNAC supplemented diet (OG, *n* = 5). The OC mice were fed a regular-chow diet (as described above, with protein 25.6%, 3.0 kcal/g feed; Harlan Teklad, Indianapolis, IN, USA) for eight-weeks, and the OG mice were pair-fed to the OC mice with the GlyNAC-supplemented diet (as described above, with protein 23.5%, 3.0 kcal/g feed, N-acetylcysteine (NAC) 1.6 mg/g feed and glycine 1.6 mg/g feed; Harlan Teklad, Indianapolis, IN, USA) for eight-weeks. Because they were pair-fed and the OG mice ate all of their feed daily, both groups consumed identical calories and protein nitrogen per day. After completing eight-weeks on their respective diets, mice were euthanized and the heart, liver and kidneys were collected for measuring outcomes as described below.

### 2.4. Outcome Measures

#### 2.4.1. Glutathione Concentrations, Oxidative Stress and Oxidant Damage in the Heart, Liver and Kidneys

Total and reduced GSH concentrations were measured by ultraperformance liquid chromatography (Waters ACQUITY UPLC System) as reported by us previously [29,30], and concentrations of oxidized glutathione (GSSG) were calculated as the difference between total and reduced GSH. Oxidative stress (OxS) was measured using the TBARS kit (Cayman Chemical, Ann Arbor, MI, USA).

#### 2.4.2. Protein Isolation and Immunoblot Analyses

Standard techniques were used as reported by us previously [29]. Heart, liver and kidney tissues from Y, OC and OG mice were used with 40 µg protein lysate per lane. Three sets were done for each blot, with one mouse from each group of Y, OC and OG mice per set. The tissue samples were lysed in 1 × lysis buffer (20 mM Tris-HCL [pH 7.5], 150 mM NaCl, 1 mM Na2EDTA, 1 mM EGTA, 1% Triton X-100, 2.5 mM sodium pyrophosphate, 1 mM b-glycerophosphate, 1 mM Na3VO4, 1 mg/mL leupeptin) obtained from Cell Signaling Technology, supplemented with protease inhibitor 1 mM PMSF [29]. Samples were freeze-thawed followed by centrifugation at 14,000 rpm for 30 min at 4 °C. Protein concentration was measured using a Pierce^TM^ BCA Protein Assay Kit (Thermo Scientific). Equal amounts of protein lysate were separated by SDS-PAGE and transferred to a polyvinylidene difluoride membrane (PVDF) membrane (Thermo Scientific). The membranes were incubated in 5% *w*/*v* skimmed milk for 1h at room temperature followed by primary antibodies at 4 °C overnight. The primary antibodies used in this study included glutamate cysteine ligase catalytic subunits (GCLC), glutamate cysteine ligase modifier subunit (GCLM), glutathione synthetase (GSS), peroxisome proliferator-activated receptor-gamma coactivator (PGC)–1alpha (PGC1α), peroxisome proliferator-activated receptor α (PPARα), mitochondrial ATP synthase F1 subunit alpha (ATP5A), PTEN-induced kinase 1 (PINK1); sirtuin-3 (SirT3), and phospho-H2A histone family member X (pH2AX). The secondary antibody conjugated with horseradish peroxidase was added for 1h at room temperature. Membranes were washed with 1X tris buffered saline with Tween^®^20 (TBST) and developed using SuperSignal^TM^ West Dura Extended Duration Substrate (Thermo Scientific) on autoradiography film. The optical density was quantified using grayscale measurements in ImageJ 1.51j8 software (NIH) and normalized to the loading control β-actin.

### 2.5. Statistical Analyses

Data are expressed as means ± SEM. A repeated measures analysis of variance (ANOVA) with Tukey’s multiple comparison test was used to compare results in young mice (Y) vs. old control mice (OC) vs. old mice supplemented with GlyNAC (OG) for total glutathione in the heart, liver and kidneys. A similar ANOVA analysis was used to compare reduced glutathione, oxidized glutathione and TBARS in these mice for the same organs. A Student’s *t*-test was used to compute differences in means between the length of life of control and GlyNAC supplemented mice, and for western blot analyses. Statistical analyses were performed with GraphPad Prism version 8.0 (GraphPad Software, San Diego, CA, USA), and results are considered to be statistically significant at *p* < 0.05.

## 3. Results

### 3.1. Study 1: The Effect of GlyNAC Supplementation on Longevity

**Figure 1:**
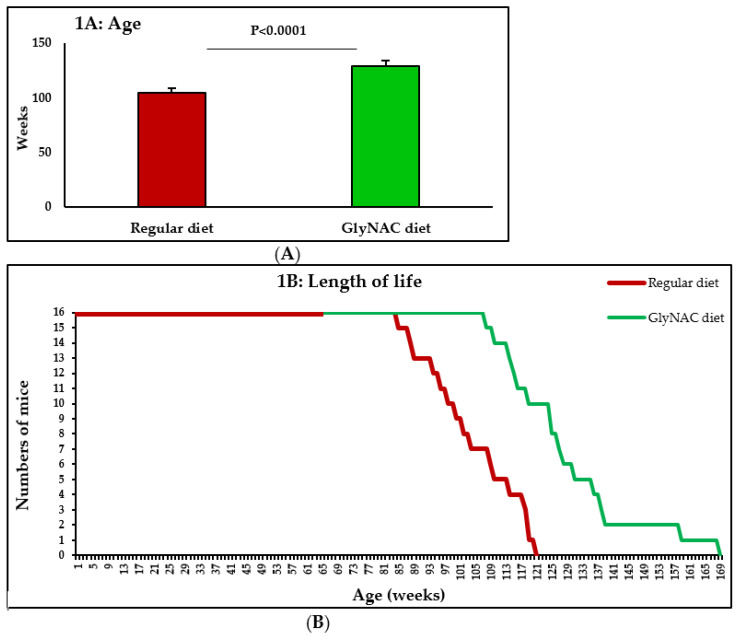
The effect of supplementing an isocaloric-isonitrogenous regular diet vs. GlyNAC-supplemented diet on length of life in C57BL/6J mice from the age of 65-weeks. (**A**) Mean age; (**B**) Length of natural life. Data shown as mean ± SE (standard error).

Compared to control mice consuming a regular diet ad libitum, mice receiving the GlyNAC supplemented diet *ad libitum* increased their length of life by 23.7% (104.0 ± 3.0 vs. 128.6 ± 4.2 weeks, *p* < 0.0001), and this was similar in both genders (males 24.2% increase; females 23.4% increase) (Figure 1A,B).

### 3.2. Study 2: The Effect of GlyNAC Supplementation on GSH, OxS, Mitochondrial Dysfunction, Mitophagy, Nutrient Sensing and Genomic Damage in the Heart, Liver and Kidneys of Old Mice

#### 3.2.1. GSH Concentrations

**Figure 2:**
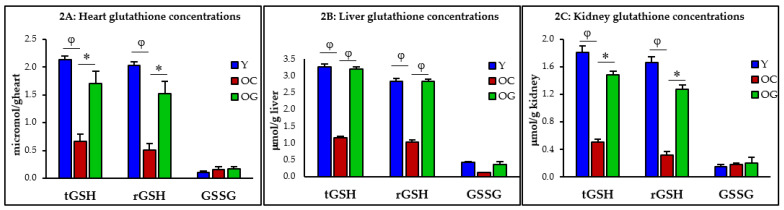
Glutathione concentrations in the heart (**A**), liver (**B**) and kidneys (**C**) in five mice/group of young mice (Y), old control mice on the regular diet (OC) and old mice consuming the GlyNAC supplemented diet (OG). t-GSH, r-GSH and GSSG = total, reduced and oxidized glutathione concentrations. * = *p* < 0.05; φ = *p* < 0.01. Results are reported as mean ± SE (standard error).

Compared to young mice, the concentrations of total-GSH in old control mice were 69% (*p* < 0.001), 65% (*p* < 0.0001) and 72% (*p* < 0.01) lower in the heart, liver and kidneys, and concentrations of reduced-GSH concentrations were 75% (*p* < 0.05), 64% (*p* < 0.0001) and 81% (*p* = 0.01) lower in the same organs, respectively. When GlyNAC supplemented old mice were compared to old mice receiving the regular diet, their total-GSH concentrations were higher by 156% (*p* < 0.05), 177% (*p* < 0.0001) and 193% (*p* < 0.05) in the heart, liver and kidneys, and reduced-GSH concentrations were higher by 204% (*p* < 0.01), 174% (*p* < 0.0001) and 301% (*p* < 0.05) in the same organs (Table 1, Figure 2A–C).

#### 3.2.2. GSH Synthesis

**Figure 3:**
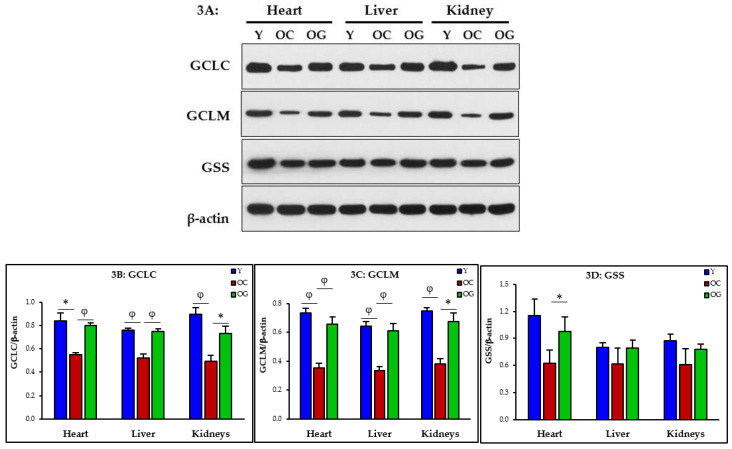
(**A**–**D**). Western blots for protein expression; each blot represents one set from the heart, liver and kidneys of three mice/group of young mice (Y), old control mice on the regular diet (OC) and old mice consuming the GlyNAC supplemented diet (OG). GCLC and GCLM = Glutamate cysteine ligase, catalytic and modifier subunits; GSS = Glutathione Synthetase. Quantification of Immunoblots of GCLC, GCLM and GSS in the heart, liver and kidneys. Optical density of protein expression was normalized to the loading control (β-actin). * = *p* < 0.05; φ = *p* < 0.01. Results are reported as mean ± SE (standard error).

GSH synthesis was assessed as the expression of the enzymes of GSH synthesis in the heart, liver and kidneys. These include the catalytic and modifier subunits of glutamate-cysteine ligase, the key rate limiting enzyme of the first step GSH synthesis (GCLC, GCLM), and glutathione synthetase (GSS, the enzyme catalyzing the final step of GSH synthesis). Compared to young mice, old control mice (on the regular diet) had significantly deficient expression of GCLC and GCLM in the heart (*p* < 0.05; *p* < 0.01), liver (*p* < 0.01; *p* < 0.01) and kidneys (*p* < 0.01; *p* < 0.01), and GlyNAC-supplementation improved/corrected the expression of GCLC and GCLM in the heart (*p* < 0.01; *p* < 0.01), liver (*p* < 0.01; *p* < 0.01) and kidneys (*p* < 0.05; *p* < 0.05), and improved expression of GSS in the heart (*p* < 0.05). These data suggest that deficient synthesis contributes to GSH deficiency, and can be corrected with GlyNAC supplementation (Figure 3A–D).

#### 3.2.3. Oxidative Stress

**Figure 4:**
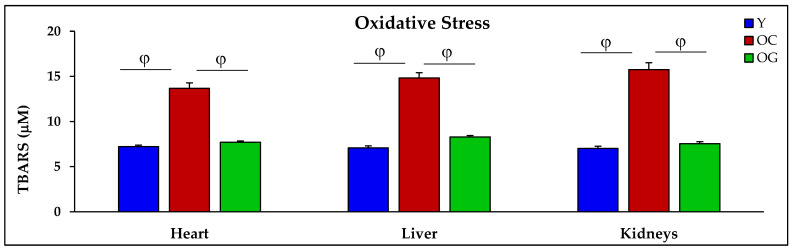
Oxidative stress (as concentrations of TBARS) in the heart, liver and kidneys of five mice/group of young mice (Y), old control mice on the regular diet (OC) and old mice consuming the GlyNAC supplemented diet (OG). φ = *p* < 0.01. Results are reported as mean ± SE (standard error).

Compared to young mice, in old control mice the concentrations of TBARS (marker of OxS) were higher in the heart, liver and kidneys by 89.7% (*p* < 0.01), 109.3% (*p* < 0.001) and 124.4% (*p* < 0.001) respectively. Compared to the control old mice, the GlyNAC supplemented old mice had lower TBARS concentrations in the heart, liver and kidneys by 43.7% (*p* < 0.01), 44.2% (*p* < 0.001) and 52.2% (*p* < 0.001), respectively (Table 1, Figure 4).

#### 3.2.4. Mitochondrial Dysfunction

**Figure 5:**
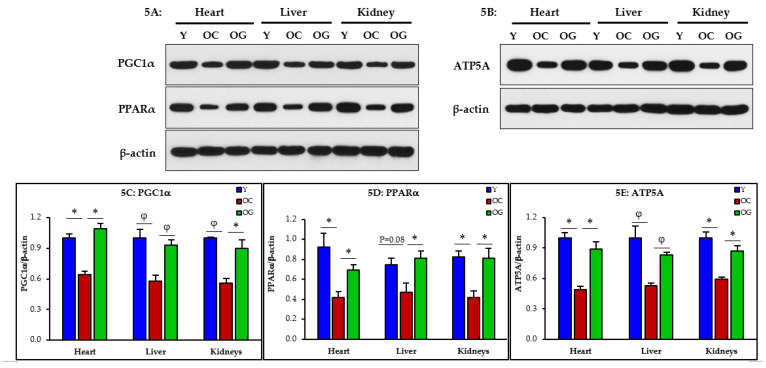
(**A–E**) Western blots for protein expression; each blot represents one set from the heart, liver and kidneys of three mice/group of young mice (Y); old control mice on the regular diet (OC); old mice consuming the GlyNAC supplemented diet (OG). PGC1α= PPARG coactivator 1 alpha; PPARα= Peroxisome proliferator-activated receptor α; ATP5A= mitochondrial ATP synthase. Quantification of Immunoblots of PGC1α, PPARα, and ATP5A in the heart, liver and kidneys: optical density of protein expression was normalized to the loading control (β-actin). * = *p* < 0.05; φ = *p* < 0.01. Results are reported as mean ± SE (standard error).

Mitochondrial function assessed by western blots showed that compared to young mice, old control mice had significantly lower expression in the heart, liver and kidneys of key regulators of mitochondrial biogenesis, energy metabolism and ATP synthesis measured as PGC1α (heart *p* < 0.05; liver *p* < 0.01; kidneys *p* < 0.01), PPARα (heart *p* < 0.05; liver *p* = 0.08; kidneys *p* < 0.05) and ATP5A (heart *p* < 0.05; liver *p* < 0.001; kidneys *p* < 0.05) indicating the presence of mitochondrial dysfunction. Compared to the control old mice, the GlyNAC supplemented old mice had significantly higher expression of PGC1α (heart *p* < 0.05; liver *p* < 0.01; kidneys *p* < 0.05), PPARα (heart *p* < 0.05; liver *p* < 0.05; kidneys *p* < 0.05) and ATP5A (heart *p* < 0.05; liver *p* < 0.01; kidneys *p* < 0.05) indicating reversal of mitochondrial dysfunction in these organs (Figure 5A–E).

#### 3.2.5. Mitophagy

**Figure 6:**
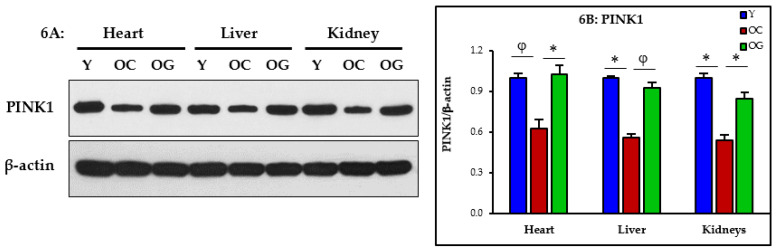
(**A**) Western blots for protein expression; each blot represents one set from the heart, liver and kidneys of three mice/group of young mice (Y); old control mice on the regular diet (OC); old mice consuming the GlyNAC supplemented diet (OG). PINK1= PTEN-induced kinase 1. (**B**) Quantification of Immunoblots in the heart, liver and kidneys: optical density of protein expression was normalized to the loading control (β-actin). * = *p* < 0.05; φ = *p* < 0.01. Results are reported as mean ± SE (standard error).

Mitophagy was assessed as western blot expression of PINK1, and was significantly lower in the heart (*p* < 0.01), liver (*p* < 0.05) and kidneys (*p* < 0.05) of old mice compared to young mice, indicating impaired clearance of damaged or nonfunctional mitochondria. Compared to the control old mice, the GlyNAC supplemented old mice had significantly improved PINK1 expression in the heart (*p* < 0.05), liver (*p* < 0.01) and kidneys (*p* < 0.05) indicating improved mitophagy (Figure 6A,B).

#### 3.2.6. Nutrient Sensing

**Figure 7:**
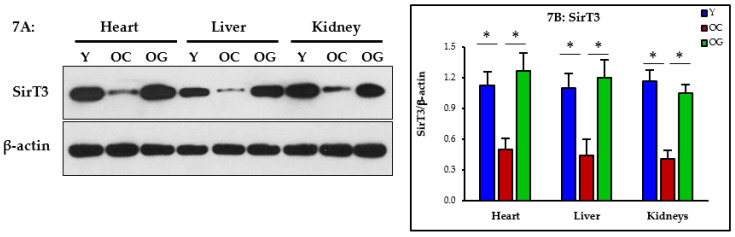
(**A**) Western blots for protein expression; each blot represents one set from the heart, liver and kidneys of three mice/group of young mice (Y); old control mice on the regular diet (OC); old mice consuming the GlyNAC supplemented diet (OG). SirT3 = Sirtuin 3. (**B**) Quantification of Immunoblots in the heart, liver and kidneys: optical density of protein expression was normalized to the loading control (β-actin). * = *p* < 0.05. Results are reported as mean ± SE (standard error).

Nutrient sensing was assessed as the expression of Sirtuin-3 (SirT3) in the heart, liver and kidneys. Compared to young mice, the old control mice had significantly lower expression of SirT3 in the heart (*p* < 0.05), liver (*p* < 0.05) and kidneys (*p* < 0.05) indicating impaired nutrient sensing in vital organs of the body. Compared to the control old mice, the GlyNAC supplemented old mice had significantly higher SirT3 expression in the heart (*p* < 0.05), liver (*p* < 0.05) and kidneys (*p* < 0.05), indicating restoration of nutrient sensing (Figure 7A,B).

#### 3.2.7. Genomic Damage

**Figure 8:**
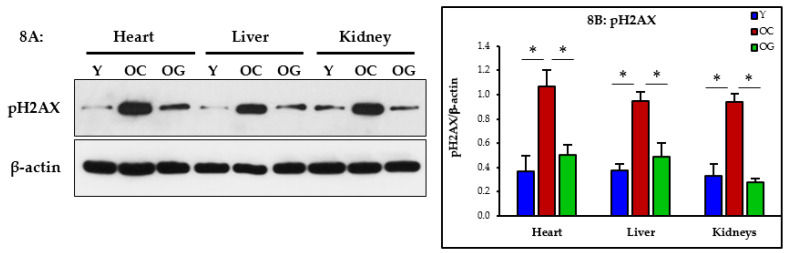
(**A**) Western blots for protein expression; each blot represents one set from the heart, liver and kidneys of three mice/group of young mice (Y); old control mice on the regular diet (OC); old mice consuming the GlyNAC supplemented diet (OG). (**B**) Quantification of immunoblots in the heart, liver and kidneys: optical density of protein expression was normalized to the loading control (β-actin). * = *p* < 0.05. Results are reported as mean ± SE (standard error).

The assessment of the western blot expression of phosphorylated H2AX (pH2AX) showed significantly higher expression in the heart (*p* < 0.05), liver (*p* < 0.05) and kidneys (*p* < 0.05) of old control mice compared to young mice, indicating greater genomic damage. Compared to control old mice, the GlyNAC supplemented old mice had significantly lower pH2AX expression in the heart (*p* < 0.05), liver (*p* < 0.05) and kidneys (*p* < 0.05), indicating a decrease in genomic damage in these organs (Figure 8A,B).

## 4. Discussion

The salient discoveries reported in this manuscript are that (a) GlyNAC supplementation in C57BL6J mice increases length of life by 24%; (b) compared to young mice, the heart, liver and kidneys of old mice had deficient GSH synthesis and GSH concentrations, elevated OxS and genomic damage, mitochondrial dysfunction, and impaired mitophagy and nutrient sensing; (c) GlyNAC supplementation corrects these defects in the heart, liver and the kidneys of old mice. pH2AX/β-actin

### 4.1. GlyNAC Supplementation Increases Longevity

This manuscript reports that that supplementing GlyNAC in wild-type mice from a relatively younger age of 65-weeks significantly increased length of life (in both genders) by 24%. Elevated OxS and mitochondrial dysfunction are two well-known cardinal defects responsible for the aging process [2,3], and these defects universally affect all cells and organs in the body [4,5,6,7,8,9,10,11,12,13,14,15,16,17,18,19]. Additional age-associated defects such as elevated genomic damage, and abnormalities in mitophagy and nutrient sensing were also identified as defects contributing to the aging process [20]. In prior human trials studying aging and premature aging, we found and reported that supplementing GlyNAC improved/reversed GSH deficiency, OxS, mitochondrial dysfunction, abnormal mitophagy, nutrient sensing and genomic damage [29,30]. Because these defects contribute to aging, we hypothesized that preventing or attenuating the development of these defects during the aging process could prolong length of life. Our studies found that supplementing GlyNAC significantly increases length of life and corrects these key defects linked to the aging process in multiple vital organs. These findings are discussed below.

Why was this study done in mice, and not humans? An obstacle to conducting human clinical trials to test the effect of an intervention on length of life is limited by the duration of time it would take to confirm or refute the intervention being tested, and studying rodents or other models can practically provide a proof-of-concept because they can be done in a time-sensitive manner. Our findings on the impact of GlyNAC on specific defects in humans (as reported in our published human trials [29,30]) have important implications for longevity, but to prove that GlyNAC could increase length of life required confirmation in a rodent study. We have shown previously in a study in HIV-patients that GlyNAC supplementation could be beneficial for reversing premature aging by correcting GSH deficiency, OxS, mitochondrial dysfunction, mitophagy, nutrient sensing and genotoxicity, and resulted in improved muscle strength, body composition and cognition, and that these benefits receded after stopping GlyNAC [29]. We reported that similar defects exist in older adults (OA) in terms of GSH deficiency, increased OxS, mitochondrial dysfunction and genomic damage, and that a longer duration of supplementation with GlyNAC for 24-weeks improved/reversed these defects, and that these benefits receded on stopping GlyNAC supplementation [30]. The results of these human trials suggest that GlyNAC supplementation combats these age-associated defects and prevents premature aging. The results of the rodent studies in this manuscript complement the findings of these human studies by showing the reversal of these defects in three vital organs of the body, and extension of life by 24%.

### 4.2. GlyNAC Supplementation Corrects Age-Associated Glutathione Deficiency, Oxidative Stress and Oxidant Damage

We reported earlier that GSH deficiency in aging occurs due to diminished GSH synthesis [31], and was associated with elevated OxS. GlyNAC supplementation for two weeks corrected GSH synthesis, improved GSH concentrations and lowered OxS [31]. In a recently reported open-label human clinical trial, we found that OA supplemented with GlyNAC for a longer duration of 24-weeks improved intracellular RBC-GSH concentrations to levels seen in young adults, and that stopping GlyNAC for 12-weeks led to a fall in RBC-GSH concentrations to pre-supplementation values [30]. In old C57BL/6 mice we found impaired GSH synthesis and low GSH concentrations in the skeletal muscle and liver together with elevated OxS, and these defects corrected with GlyNAC supplementation, and not isocaloric-isonitrogenous placebo) [28]. These data suggest the presence of generalized GSH deficiency due to impaired GSH synthesis in aging. In this manuscript we report the effect of GlyNAC supplementation on GSH synthesis and concentrations in the heart, liver and kidneys, and this is discussed next.

#### 4.2.1. Impaired GSH Synthesis and GSH Deficiency in Aging

GSH synthesis is a two-step process where in the first step cysteine first combines with glutamic acid to form the intermediate γ-glutamylcysteine catalyzed by the enzyme glutamate cysteine ligase (GCL, comprised of catalytic and modifier subunits GCLC and GCLM). In the second step catalyzed by glutathione synthetase (GSS), glycine is added to form the tripeptide glutathione (γ-glutamyl-cysteinyl-glycine). In the study reported in this paper we found that compared to young mice, the heart, liver and kidneys of old control mice had (a) lower expression of the enzymes of GSH synthesis (GCLC, GCLM and GSS); (b) lower concentrations of total and reduced glutathione, indicating that decreased synthesis is responsible for GSH deficiency with aging; (c) GlyNAC supplementation for eight-weeks corrected the expression of GCLC, GCLM and GSS, and increased GSH concentrations in all three organs, thereby correcting GSH deficiency; (d) GlyNAC supplementation did not cause an overshoot of GSH concentrations above levels seen in YA, and did not lower markers of OxS below levels in YA, suggesting that GlyNAC supplementation allows cells to successfully autoregulate cellular GSH concentrations required to combat OxS, while simultaneously avoiding reductive stress; (e) No improvements were seen in old mice receiving the isocaloric-isonitrogenous placebo diet, suggesting that increasing protein supplementation *per se* does not improve GSH synthesis or concentrations. These outcomes suggest that GlyNAC is a highly effective nutritional supplement which improves and corrects GSH deficiency in multiple vital organs in old mice, and thereby increases longevity.

#### 4.2.2. Oxidative Stress and Oxidant Damage in Aging

When in excess, ROS arising from mitochondrial respiration result in harmful and damaging OxS. The highly toxic superoxide radical (O_2_^–^) is rapidly converted to hydrogen peroxide (H_2_O_2_) by superoxide dismutase, but H_2_O_2_ is also highly reactive with cells, membranes, lipids, protein and the genome, and needs to be detoxified. GSH detoxifies the H_2_O_2_ to water by yielding its reducing equivalents, and in the process GSH is oxidized to glutathione disulfide (GSSG), (H_2_O_2_ + 2GSH → 2H_2_O + GSSG). Thus, GSH is critical for cellular protection from OxS, and GSH deficiency has been shown to result in elevated OxS in young animals [32,33]. Our study found that severely elevated OxS (as a likely consequence of GSH deficiency) in the heart, liver and kidneys of old mice improved only with GlyNAC supplementation, and not with isocaloric-isonitrogenous placebo diets. Because many age-related defects in multiple tissues are linked to OxS [4,5,6,7,8,9,10,11,12,13,14,15,16,17,18,19], the reversal of OxS in the heart, liver and kidneys of old mice in our study suggests that GlyNAC supplementation could play an important role in promoting health in aging, and thereby contributes to increasing the length of life.

### 4.3. GlyNAC Supplementation Corrects Mitochondrial Dysfunction and Impaired Mitophagy

Aging is associated with impaired mitochondrial function and defects in mitophagy [21,22,23,24], two aging hallmarks which contribute to perturbations in cellular energy availability. Because energy availability is critically necessary for life, it is possible that decreased energy availability, as an inevitable result of age-associated mitochondrial dysfunction, could limit length of life. In prior studies, we discovered and reported that GlyNAC supplementation reverses mitochondrial dysfunction in old mice [28] and older humans [30], and in this manuscript we report that GlyNAC supplementation reversed mitochondrial dysfunction in the heart, liver and kidneys of old mice. The magnitude and severity of age-associated mitochondrial impairment in these organs becomes clear via the molecular data which shows that old mice had abnormalities in mitochondrial biogenesis and energy metabolism (PGC1α), and impaired mitochondrial β-oxidation of fatty-acids (PPARα), ATP synthesis (ATP5A), and mitophagy (PINK1). These findings indicate that there is a broad range of defects affecting mitochondrial function in aging, and together with impaired mitophagy, energy metabolism in aging is severely compromised in multiple organs. GlyNAC supplementation corrects these defects after a relatively short duration of eight-weeks. No changes occurred with an isocaloric-isonitrogenous diet, which suggests that GlyNAC is specifically necessary for benefits, and that generalized protein supplementation does not improve these defects. Because cellular processes depend on an uninterrupted energy supply to support optimal and efficient cellular function, compromised mitochondrial function will result in cellular, tissue, organ and whole-body adaptations and dysfunction commonly found in aging. Therefore, the discovery that GlyNAC supplementation corrects oxidative stress and mitochondrial defects in multiple organs in old mice suggests these as possible mechanisms whereby GlyNAC supplementation contributed to increasing longevity in old mice in our study.

### 4.4. GlyNAC Supplementation Improves Nutrient Sensing

Abnormal nutrient sensing is a hallmark of aging [20]. Sirtuins are critically important cellular nutrient sensors in the nucleus and mitochondria [34,35,36]. NAD^+^ (nicotine amide dinucleotide) is an important regulator of energy metabolism and is linked to longevity [37,38]. NAD^+^ acts in conjunction with and via sirtuin signaling. SirT3 is an important regulator of mitochondrial structural and functional proteins [39], and SirT3 adequacy is necessary for NAD^+^ action [40]. The novel discovery that GlyNAC can restore SirT3 in multiple vital organs has important implications for improving health, longevity and NAD^+^-based biology in aging.

### 4.5. GlyNAC Supplementation Reverses Genomic Damage

Chromosomal DNA are damaged by internal and external forces [41,42]. The old mice in this study had elevated genomic DNA damage (pH2AX) which improved with GlyNAC supplementation. Because genomic damage is a hallmark of aging [20], it is possible that correcting genomic damage could have contributed to increasing length of life.

### 4.6. Connecting the Dots

An uninterrupted supply of energy is necessary to sustain cellular function, organ health and maintain life. Mitochondria act in coordination with nutrient sensors in the generation of cellular energy, and their regulation is evolutionarily conserved and linked to longevity. The process of mitochondrial energy generation also generates reactive oxygen species (ROS), and excess ROS results in harmful and destructive OxS which damage mitochondria. Thus, mitochondrial dysfunction and OxS are two sides of the same coin with one leading to the other. Maintaining a harmonious balance between preventing cellular OxS and promoting optimal cellular mitochondrial function is the function of cellular GSH. We have shown that inducing GSH deficiency results in mitochondrial dysfunction [28], and that supplementing GlyNAC corrects all three defects (i.e., GSH deficiency, OxS and mitochondrial dysfunction) [28,29,30]. While lifespan is finite in all species, could the combination of key defects such as GSH deficiency, elevated OxS, and mitochondrial dysfunction limit length of life via their influence on cellular processes? OxS has been linked to all-cause mortality in human population studies [43,44], and mitochondrial dysfunction and autophagy have been linked to pathways affecting longevity [45,46]. Collectively, these data suggest that reversing or attenuating oxidative stress and mitochondrial dysfunction could potentially increase lifespan. GSH is strongly conserved evolutionarily across life from unicellular organisms to multiple species, including humans [47,48,49] and we suggest that the combined effect of glycine, NAC and GSH (the ‘power of 3′) plays an important and essential role in determining lifespan. We reported earlier that supplementing GlyNAC improves these defects in humans [29,30,31]. From unpublished studies of mice in our lab, we found that GSH deficiency tends to occur in C57BL/6J mice after the age of approximately 65-weeks of age. Therefore, in this study we supplemented C57BL/6J mice with GlyNAC from the age of 65-weeks for the rest of their natural life, and found that this significantly increased their length of life by about 24%. Why did this happen? As discussed above, the answers could lie in correcting a combination of defects including GSH deficiency, OxS, mitochondrial dysfunction, impaired mitophagy, abnormal nutrient sensing, and genomic damage, with the latter four defects being hallmarks of aging. In this manuscript we have presented evidence that GlyNAC supplementation results in the reversal of these defects in three vital organs (heart, liver and kidneys). In earlier publications we have reported that GlyNAC supplementation improves cardiac function [50], lowers liver fat content [28,29], and improves renal function [29,30]. In published human trials we reported that GlyNAC supplementation has the potential to reverse premature aging and improve muscle strength, exercise capacity, insulin resistance, cognition, blood pressure, body composition and quality of life [29,30,31,51,52,53]. GlyNAC supplementation also improves cognition, as reported in our prior studies in older humans and HIV-infection [29,30]. These studies suggest that GlyNAC could improve the health of multiple organ systems including the brain and the nervous system, and needs investigation in future studies. Immune health and immunosenescence are reported to contribute to the morbidity and mortality of the elderly [54], and although the effect of GlyNAC on immune health was not evaluated in our study, this should be addressed in future studies. The data in this manuscript provides proof-of-concept that GlyNAC supplementation could significantly increase length of life. When the findings in our prior GlyNAC studies [29,30,31,51,52,53] are seen together with the new data reported in this manuscript, GlyNAC emerges as a highly effective nutritional supplement with the potential to improve both healthspan and lifespan to improve healthy aging, and health in aging. These data support the need for more research on GlyNAC supplementation in aging.

### 4.7. Why GlyNAC Works

Aging has been linked to glycine and cysteine deficiencies [55,56], and we reported earlier that supplementing GlyNAC corrects both these defects in older humans [31]. Glycine is an important 1-carbon donor and a methyl-group donor for purine synthesis required for DNA, functions as a brain neurotransmitter, and supports multiple cellular reactions. Cysteine is a sulfhydryl group donor which is required for mitochondrial energy generation (for example, mitochondrial co-enzyme A (CoA-SH) requires a sulfhydryl group for its function), and is necessary for multiple cellular processes and reactions. Glycine and cysteine combine to form GSH, a master antioxidant which is evolutionarily conserved across species, and is critically necessary for cellular and mitochondrial health, and could play a role in cellular signal transduction pathways. We have termed the combination of glycine, cysteine and GSH as the ‘power of three’, as all three components combine to promote healthy aging [57].

It is important to recognize that cells require a small amount of reactive oxygen species for normal function, as these participate in cell signaling [58]. Excessive depletion of reactive oxygen species can result in reductive stress, a harmful condition which can result in cell and organ damage [59,60]. This is the central risk associated with the consumption of exogenous antioxidants to target cellular oxidative stress because the dosimetry is unknown (as every organ generates different amounts of ROS, and maintains different amounts of GSH in a dynamic and rapid changing cellular redox *milieu*). This manuscript provides evidence for the existence of these variations in the heart, liver and kidneys of aged mice. Under physiological conditions, cells have to perform a delicate balancing act of preventing excess accumulation of ROS while simultaneously preventing excess depletion of ROS, and these demands could be exacerbated by disease or abnormalities associated with aging. GlyNAC differs from other exogenous antioxidants because it does not attempt to override the natural ability of cells to combat OxS. Instead, GlyNAC provides glycine and cysteine to support cellular GSH synthesis and concentrations, and supports the cellular ability to autoregulate its redox *milieu* in every organ and tissue. In this manuscript it can be clearly seen that GlyNAC increases GSH and lowers OxS in the heart, liver or the kidneys to match controls, and that GSH concentrations do not exceed control values—this is also seen in all our human clinical trials supplementing GlyNAC [29,30,31,50,51,52,53]. Collectively, these studies provide evidence to support the safety and efficacy of GlyNAC in providing cellular protection, correcting key cellular defects, maintaining mitochondrial health, and ultimately promoting healthspan and lifespan.

### 4.8. GlyNAC Is Not the Same as NAC-Alone or GSH-Alone

(a) GSH synthesis requires both cysteine (from NAC) and glycine. Supplementing NAC-alone does not provide glycine, and only GlyNAC provides both the glycine and cysteine needed for GSH synthesis. Proof to support this point comes from data in this study which shows that GlyNAC supplementation improves GSH synthesis and GSH concentrations to correct GSH deficiency in multiple vital organs of the body. No study has reported extension of mammalian life with NAC-alone and, on the contrary, supplementing NAC-alone in *C. elegans* was found to accelerate aging and shorten lifespan [61]. However, as reported in this manuscript, GlyNAC supplementation in mice extends life.

(b) As discussed above, cells need to combat OxS and simultaneously avoid reductive stress. As seen in this report, the heart, liver and the kidneys have differing GSH concentrations. Indeed, every organ of the body maintains its own GSH concentrations based on their metabolic activity, generation of ROS and cellular needs. Exogenous compounds with antioxidant potential run into the problem of dosing—too much can induce reductive stress, and too little will not combat OxS. This is further complicated by the fact that every organ maintains a different concentration of GSH, and exogenous antioxidant dosing is unlikely to correct GSH deficiency and OxS, while simultaneously avoiding reductive stress in all organs. GlyNAC does not attempt to override or replace cellular defenses to combat OxS and reductive stress. Instead, by providing precursors to boost GSH synthesis and concentration, GlyNAC works by supporting the ability of cells to autoregulate their own GSH—thus GlyNAC can correct GSH deficiency and OxS in every cell and organ in the body, without inducing reductive stress.

(c) No studies have reported an increase in mammalian lifespan by supplementing exogenous glutathione. On the contrary, supplementation of exogenous GSH was found to accelerate aging and shorten life in *C. elegans* [61]. Because different organs maintain different concentrations of GSH, providing exogenous GSH is associated with the risk of inducing reductive stress. GlyNAC avoids this problem by allowing cells to autoregulate and maintain their required GSH homeostasis, i.e., GlyNAC does not interfere with cellular autoregulation whereby cells make the requisite amount of GSH based on cellular need. This is an important point to understand because the cellular requirement of GSH in each organ is constantly and dynamically changing and is influenced by variations in metabolic activity caused by feeding/fasting, rest/activity and waking/sleep. An additional challenge is that oral GSH is not absorbed well from the gut because it is digested and broken down into its constituent amino-acids, and in this process, cysteine is oxidized by the gut to cystine, which is not a GSH precursor. Even if GSH could be provided in a form which is absorbable, it still runs into dosing issues because cells synthesize their required GSH and do not depend on the plasma to provide GSH. For these reasons, exogenous provision of GSH is impractical and non-physiological, while GlyNAC supplementation corrects GSH deficiency and lowers OxS in vital organs, and extends life.

### 4.9. Study Limitations

Although this pilot exploratory study provides proof-of-concept that GlyNAC supplementation from a younger age can increase length of life, the main limitation is that the study was done in a relatively small number of mice, and data is not reported from every organ in the body. The positive results of this study support the need for future studies in larger numbers of mice of both genders, and evaluation of the effects in more organs in male and female mice to determine possible gender differences.

## 5. Conclusions

The results of these rodent studies indicate that supplementing GlyNAC increases length of life. GlyNAC supplementation also corrects GSH deficiency, OxS, and multiple aging hallmarks (mitochondrial dysfunction, abnormalities in mitophagy and nutrient sensing, and genomic damage) in vital organs such as the heart, liver and kidneys. GlyNAC supplementation could have important implications for promoting healthy aging and improving both healthspan and lifespan in humans.

## Figures and Tables

**Table 1:** Concentrations of total glutathione, reduced glutathione, oxidized glutathione (GSSG), and TBARS in the heart, liver and kidneys of five mice/group of young control mice (Y), old control mice (OC) and old mice supplemented with GlyNAC (OG). t-GSH, r-GSH and GSSG = total, reduced and oxidized glutathione concentrations. Results are reported as mean ± SE (standard error) and considered to be statistically significant at values *p* < 0.05.

	Y: Heart	OC: Heart Y vs. OC	OG: Heart OC vs. OG	Y: Liver	OC: Liver Y vs. OC	OG: Liver OC vs. OG	Y: Kidney	OC: Kidney Y vs. OC	OG: Kidney OC vs. OG
t-GSH (μmol/kg)	2.1 ± 0.1	0.7 ± 0.1 *p* < 0.001	1.7 ± 0.2 *p* < 0.05	3.3 ± 0.1	1.2 ± 0.0 *p* < 0.0001	3.2 ± 0.1 *p* < 0.0001	1.8 ± 0.1	0.5 ± 0.2 *p* < 0.01	1.5 ± 0.2 *p* < 0.05
r-GSH (μmol/kg)	2.0 ± 0.1	0.5 ± 0.1 *p* < 0.01	1.6 ± 0.1 *p* < 0.05	2.9 ± 0.1	1.0 ± 0.1 *p* < 0.0001	2.8 ± 0.1 *p* < 0.0001	1.7 ± 0.1	0.3 ± 0.1 *p* < 0.01	1.3 ± 0.3 *p* < 0.05
GSSG (μmol/kg)	0.1 ± 0.0	0.2 ± 0.0 *p* = 0.5	0.2 ± 0.0 *p* = 0.9	0.4 ± 0.0	0.1 ± 0.0 *p* < 0.001	0.4 ± 0.1 *p* = 0.08	0.1 ± 0.0	0.2 ± 0.0 *p* = 0.7	0.2 ± 0.0 *p* = 0.9
TBARS (μM)	7.2 ± 0.2	13.7 ± 0.6 *p* < 0.01	7.7 ± 0.1 *p* < 0.01	7.1 ± 0.2	14.8 ± 0.6 *p* < 0.001	8.3 ± 0.2 *p* < 0.001	7.0 ± 0.2	15.8 ± 0.8 *p* < 0.001	7.5 ± 0.2 *p* < 0.001

## Data Availability

All relevant data are contained in this manuscript.
